# Awakening dormant neurons long after spinal cord injury

**DOI:** 10.1371/journal.pbio.3001830

**Published:** 2022-09-29

**Authors:** Mary L. Tapia, Kevin K. Park

**Affiliations:** Department of Neurological Surgery, The Miami Project to Cure Paralysis, University of Miami Miller School of Medicine, Miami, Florida, United States of America

## Abstract

Neurons lack the ability to regenerate after injury. This Primer explores a new Preregistered Article in PLOS Biology that found that pharmacologically boosting regenerative capacity long after injury in mice, together with an enriched animal environment, promotes axonal and synaptic plasticity.

## Teaser

Neurons lack the ability to regenerate after injury. A new Preregistered Article in *PLOS Biology* finds that pharmacologically boosting regenerative capacity long after injury in mice, together with an enriched animal environment, promotes axonal and synaptic plasticity.

The estimated annual incidence of traumatic spinal cord injury (SCI) is approximately 54 cases per 1 million people in the United States, which equals about 18,000 new cases of SCI each year [1]. According to the National Spinal Cord Injury Statistical Center, approximately 300,000 people are living with SCI in the United States alone [1,2]. In general, neurons in the central nervous system have limited ability to rearrange their axons (i.e., nerve fibers) and synapses after an injury. This feature restricts the reformation of lost connections, leading to limited recovery of body functions after SCI. Over the years, life expectancy in patients with SCI, as well as in the general population, has increased substantially. This increased life expectancy has been attributed not only to improved survival from initial damages, but also to improved capacity to treat secondary complications such as infections and pneumonia [2]. As people with SCI are living longer, safe regenerative strategies that can promote neuronal plasticity and ameliorate the debilitating conditions are highly sought after. In this regard, it is worth noting that compared with the acute phase of injury, neurons likely face more barriers to remodeling after prolonged SCI. For example, axons likely have undergone significant degeneration over the years, creating large distances for regrowing axons to travel. It is also likely that growth-inhibiting scars will have become consolidated around the chronic lesion site, making it more difficult for axons to grow through. Aged neurons may have further lost their natural ability to grow [3]. In fact, while significant advances have been made in promoting axon regeneration, very few studies have utilized models of chronic SCI even though the patient population with chronic SCI is most likely to benefit from axon regeneration strategies.

**Fig 1:**
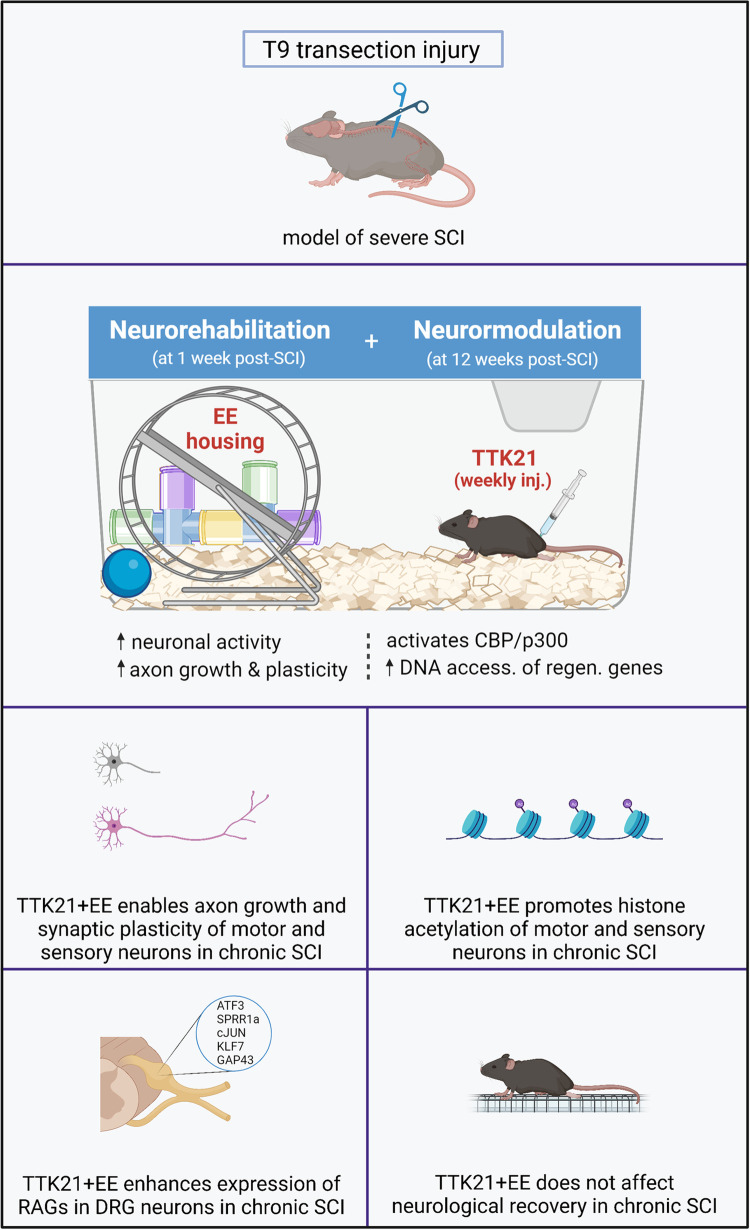
The effects of EE and delayed TTK21 treatment on neuronal plasticity and functional recovery after spinal cord injury. A drug capable of modulating DNA accessibility (i.e., TTK21) given months after injury is able to promote expression of RAGs and axonal regrowth in DRG cells and motor neurons with limited functional recovery. Created with BioRender.com. DRG, dorsal root ganglion; EE, enriched environment; RAG, regeneration associated gene.

In a new pre-registered report, Müller and colleagues asked whether combining epigenetic-driven stimulation of regenerative gene expression in mice with an enriched environment (EE) could improve regeneration long after SCI had occurred [4] (Fig 1). In the past decade, numerous studies have identified genes and gene products that contribute to the limited regenerative capacity of neurons [5]. Researchers have also begun to unravel epigenetic mechanisms (changes in gene activity that do not involve changes in DNA sequence) that control the expression of these genes [6]. For example, studies have revealed that genetic or pharmacological activation of epigenetic modulators (e.g., P300 and CBP) allows DNA strands to be more “accessible,” increases expression of distinct sets of genes, and enhances axon regeneration [7,8]. This is truly remarkable, considering that epigenetic modulators can alter the expression of hundreds of genes, only some of which are bona fide regeneration-promoting genes. Importantly, knowledge gained from these studies has led to the discovery of drugs that can promote axonal rearrangement and regeneration in animal models of SCI [8]. These findings have raised hopes of one day being able to treat patients with SCI. However, the approaches used to date have faced significant challenges, and they generally have not resulted in robust improvement of behavioral functions such as forelimb use and locomotion.

In their study, Müller and colleagues [4] provided EE housing for the injured animals, in which the animals’ cages contained toys, tunnels, running wheels, and enriched bedding. This housing condition is quite different to the conventional animal housing provided in most SCI research laboratories, which often includes only the enriched bedding and, in some cases, tunnels. Considering that humans are usually encouraged to engage in physical activities following an SCI, EE perhaps better reflects the “physiological” conditions experienced by humans with the condition. Notably, an increase in neuronal activity, either associated with EE or direct electrical stimulation soon after SCI, can increase axon growth and new neuronal connections, leading to improvement in functional recovery [9]. Intriguingly, neuronal activity not only promotes the expression of regeneration-promoting genes but also many other cellular processes beneficial for neuronal regeneration, such as enhancement of mitochondrial function and axonal transport among others [9]. These observations raise an obvious question of whether concomitant provision of EE and the drugs known to increase the expression of regeneration-promoting genes provides a synergistic benefit.

In combination with EE, Müller and colleagues evaluated whether a drug called TTK21 can enhance axonal plasticity and behavioral recovery when it is administered months after SCI. TTK21 is a drug known to activate P300 and CBP (i.e., promoters of DNA accessibility) and to allow expression of many genes beneficial to regeneration [8]. They found that TTK21 promotes induction of beneficial genes for regeneration in the CNS neurons and considerable regrowth of axons around the lesion area. However, these treatments failed to promote regeneration of injured axons throughout the lesion site, indicating that the lesion environment is a formidable barrier for these axons at the chronic stage. Importantly, the authors have found that despite the signs of neuronal plasticity, the behavioral functions (e.g., stepping on grid walk) were not improved in these animals. They conclude that in chronic SCI, it is feasible to use epigenetic modulators and enhance axon regrowth, at least to some extent. These results further support previous studies that have shown that deleting certain genes in neurons long after SCI can promote axon regeneration [10]. However, to attain meaningful functional recovery, it has become clear that additional strategies are needed including those that can make the injury site less inhibitory to axon growth.

Much progress has been made in identifying strategies to augment the ability of neurons to regrow axons. The new study in *PLOS Biology* underscores the power of epigenetic modification and the feasibility of “awakening” dormant neurons, even for animals that have been injured for a long time [4]. Considering that there are hundreds and thousands of patients living with chronic SCI, a key question remains as to whether drugs such as TTK21 will induce recovery when it is given in combination with stem cell or biomaterial grafts known to neutralize the growth inhibitory injury site [11,12].
